# Halofuginone inhibits phosphorylation of SMAD-2 reducing angiogenesis and leukemia burden in an acute promyelocytic leukemia mouse model

**DOI:** 10.1186/s13046-015-0181-2

**Published:** 2015-06-23

**Authors:** Patricia A. Assis, Lorena L. De Figueiredo-Pontes, Ana Silvia G. Lima, Vitor Leão, Larissa A. Cândido, Carolina T. Pintão, Aglair B. Garcia, Fabiano P. Saggioro, Rodrigo A Panepucci, Fernando Chahud, Arnon Nagler, Roberto P. Falcão, Eduardo M. Rego

**Affiliations:** Hematology and Oncology Divisions of the Department of Internal Medicine, Medical School of Ribeirão Preto, University of São Paulo, Ribeirão Preto, SP 14049900 Brazil; Pathology Department, Medical School of Ribeirão Preto, University of São Paulo, Ribeirão Preto, SP 14049900 Brazil; Hematology Division and Cord Blood Bank, Chaim Sheba Medical Center, Tel Aviv University, Tel Hashomer, 6997801 Israel

**Keywords:** Acute Promyelocytic Leukemia, Halofuginone, SMAD, TGF-β, VEGF, Angiogenesis

## Abstract

**Background:**

Halofuginone (HF) is a low-molecular-weight alkaloid that has been demonstrated to interfere with Metalloproteinase-2 (MMP-2) and Tumor Growth Factor-β (TGF-β) function and, to present antiangiogenic, antiproliferative and proapoptotic properties in several solid tumor models. Based on the fact that high levels of Vascular Endothelial Growth Factor (VEGF) and increased angiogenesis have been described in acute myeloid leukemia and associated with disease progression, we studied the *in vivo* effects of HF using an Acute Promyelocytic Leukemia (APL) mouse model.

**Methods:**

NOD/SCID mice were transplanted with leukemic cells from hCG-PML/RARA transgenic mice (TM) and treated with HF 150 μg/kg/day for 21 days. The leukemic infiltration and the percentage of VEGF+ cells were evaluated by morphology and flow cytometry. The effect of HF on the gene expression of several pro- and antiangiogenic factors, phosphorylation of SMAD2 and VEGF secretion was assessed *in vitro* using NB4 and HUVEC cells.

**Results:**

HF treatment resulted in hematological remission with decreased accumulation of immature cell and lower amounts of VEGF in BM of leukemic mice. *In vitro,* HF modulated gene expression of several pro- and antiangiogenic factors, reduced VEGF secretion and phosphorylation of SMAD2, blocking TGF-β-signaling.

**Conclusion:**

Taken together, our results demonstrate that HF inhibits SMAD2 signaling and reduces leukemia growth and angiogenesis.

**Electronic supplementary material:**

The online version of this article (doi:10.1186/s13046-015-0181-2) contains supplementary material, which is available to authorized users.

## Background

Halofuginone (HF) is a low-molecular-weight quinazolinone alkaloid that has been demonstrated to present potent antitumor effects due antiproliferative [1, 2] and proapoptotic [3] properties. Treatment with HF impaired proliferation and induced apoptosis of several multiple myeloma cell lines and showed antitumor activity in a myeloma xenograft mouse model [3]. In addition, HF antiangiogenic properties also have been shown to contribute to control of tumor growth [4–6]. In a rat brain tumor model, HF treatment markedly reduced vascularization and vessel maturation resulting in reduced tumor burden and metastasis [6].

Angiogenesis is one of the most important mechanisms of tumor progression [7, 8] and has been associated to development of hematological malignancies [9]. Several studies have reported an increase in bone marrow (BM) microvessel density (MVD) and high VEGF (Vascular Endothelial Growth Factor) expression in multiple myeloma [10], non-Hodgkin lymphoma [11], and acute leukemias [12, 13]. Particularly, in acute myeloid leukemia (AML), high levels of VEGF and increased MVD were found in leukemic BM biopsies [12] and correlated with lower rates of complete remission and lower overall survival [14]. In a study analyzing 12 BM biopsies from patients with acute promyelocytic leukemia (APL), Kini *et al.* [15] detected increased MVD associated with high VEGF expression. After standard treatment with ATRA (all-*trans* retinoic acid), VEGF levels and BM MVD were normalized. Another clinical study with 17 new cases of APL associated the efficacy of the arsenic trioxide treatment with its antiangiogenic effect, demonstrated by the reduced MVD in the BM and increased survival [16].

Despite the fact that all-trans retinoic acid (ATRA) is largely applied for the treatment of APL, the use of ATRA alone does not achieve long-term remission [17, 18]. Thus, associations of ATRA with chemotherapy or arsenic trioxide have been more successful in improve long-term survival [19]. However, these treatment regiments have limited application for elderly patients or those with serious heart conditions [20]. In addition, the therapy with arsenic trioxide is not available in several developing countries [21] and the 10-20 % of patients treated with ATRA-chemotherapy or ATRA-arsenic trioxide association will relapse [22, 23]. Therefore, the development of new strategies for APL therapeutics is necessary and angiogenesis is a potential target to achieve a better outcome.

The exact mechanism by which HF impairs angiogenesis has not yet been fully characterized. Elkin *et al.* [24] reported that HF treatment led to the reduction in metalloproteinase-2 (MMP-2) expression and inhibition of bladder carcinoma metastasis in an animal model. However, HF effects may not be specific to malignant tissues, since the implant of silicone coated with HF resulted in reduced expression of MMP-2, bFGF and TGF-β in normal muscle [25]. In APL the TGF-β pathway is deregulated by the cytoplasmic PML isoform (cPML) [26] and we have demonstrated that HF inhibited cell proliferation and induced apoptosis in the APL cell line NB4 through modulation of TGF-β-target genes, such as c-Myc and p21 [1]. In addition, it is noteworthy to point out that the promoter region of the VEGF gene contains DNA-binding sequences for hypoxia-inducible factor 1 alpha (HIF-1α) and for the TGF-β-signaling mediators SMADs [27, 28]. Thus, activation of TGF-β pathway may be associated with increased VEGF expression and angiogenesis in acute leukemias. Here we analyzed the *in vivo* antileukemic effect of HF and its association with the modulation of proangiogenic and antiangiogenic factors using APL as a bone fide example of acute leukemia with increased angiogenesis.

## Methods

### Halofuginone

Halofuginone (HF-dl-trans-7-bromo-6-chloro-3-(3-(3-hydroxy-piperidyl)acetonyl)-(3H)-quinazolinone hydrobromide) was solubilized in lactic acid priors to dilute into phosphate buffer solution. *In vitro* ED_50_ was previous determined [1] *in vivo* doses were delineated based on previous reports of the use of this drug in solid tumors and fibrosis models [29–32].

### Mice and leukemia transplant model

BM angiogenesis was first analyzed in leukemic hCG-PML/RARA transgenic mice (TM). Notably, in this transgenic model, a lethal form of leukemia that closely resembles human APL occurs after a long pre-leukemic phase (12–15 months) and affects only 10–15 % of the TM [33]. To analyze *in vivo* effects of HF we developed an APL transplanted model. Thus, NOD/SCID mice aged 10 to 12 weeks were irradiated with 250 cGy and 24 hours later, 2 × 10^6^ viable cells from leukemic hCG-PML/RARA transgenic mice (TM) were intravenously injected. Twenty-four hours after transplantation, NOD/SCID mice were then submitted to daily treatment with 150 μg/kg/day HF (*n* = 5) or vehicle only (0.9 % NaCl; *n* = 5) as an intraperitoneal injection for 21 consecutive days. Previous experiments were performed to confirm leukemic infiltration in transplanted animals and to test the efficacy and toxicity of HF. All animal experiments using mice were conducted according to national guidelines for the care and use of laboratory animals (Brazilian College of Animal Experimentation) and were approved by the institutional Animal Experimentation Ethics Committee (protocol number 062/2008).

### Cell culture

NB4 cells, a permanent cell line harboring PML-RARα t (15;17), characterizing an APL cell line [34], was used for *in vitro* assays. Briefly, cells were cultured in RPMI 1640 with 10 % fetal calf serum (FCS, Gibco, BRL, UK). Human Umbilical Vein Endothelial Cells (HUVEC) were maintained in EBM medium supplemented with 20 % FCS [35]. All cells were maintained at 37 °C under 5 % CO_2_.

### Quantification of VEGF, angiogenin, and TGF-β

NB4 cells were treated with HF for 72 hours at doses ranging from 25 to 100 ng/mL. VEGF, angiogenin and TGF-β concentrations in supernatants were determinate by CBA (Cytometric Bead Array, BD Biosciences, San Jose, CA, USA) and/or ELISA (BD Biosciences, San Jose, CA, USA), following manufacturer’s recommendations.

### Phospho-SMAD2 (P-SMAD2) identification

The phosphorylation of SMAD2 was assessed in total protein extracts obtained from NB4 cells treated with 50, 100, and 200 ng/mL doses of HF for 6, 12, and 24 hours. We used the PathScan® Phospho-Smad2 (Ser465/467) Sandwich ELISA Kit (Cell Signaling, Danvers, MA, USA) that provides the P-SMAD2 level as a direct absorbance reason. To assess the phosphorylated protein, TGF-β1 cytokine (Sigma-Aldrich, St. Louis, MO, USA) was added to the culture (1ng/mL) one hour before the ending of HF treatment.

### Real time PCR and PCR array

mRNA expression was assessed by real time PCR. Briefly, total RNA was isolated using Trizol reagent (Invitrogen, Carlsbad, CA, USA) and was reverse transcribed using the cDNA High Capacity Archive kit (Applied Biosystems, Foster City, CA, USA). cDNA samples were used to quantify gene expression using specific primers and probes TaqMan® (Applied Biosystems, Foster City, CA, USA). Gapdh was included as endogenous internal control. PCR array was performed using the angiogenesis RT2 Profiler™ PCR Arrays kit (SA Biosciences, Frederick, MD, USA), in which each cDNA sample was processed in a 96-well plate containing 84 angiogenesis-related genes and B2M, HPRT1, RPL13A, ACTB, and GAPDH were included as endogenous internal controls in all plates. mRNA expression of non-treated cells or non-leukemic mice are expressed as Fold Change = 1. Amplification was performed in plate duplicates in a 7500 Real-Time PCR System (Applied Biosystems, Foster City, CA, USA).

### Immunohistochemistry staining for VEGF in bone marrow sections

Vertebra samples were collected from mice, fixed and serial 5-μm sections were prepared in paraffin. Immunohistochemistry was performed with primary anti-VEGF antibodies (Santa Cruz Biotechnology, Santa Cruz, CA, USA) and anti-α-actin (for TM mice) and anti-CD34 (for transplante mice) (Becton Dickinson, San Jose, CA, USA) at 1:500, 1:800 and 1:50, respectively. Peroxidase activity was revealed by 3,3′-diaminobenzidine as chromogen. Alpha-actin and CD34 expression was used for assessing microvessel density (MVD), quantified as the number of microvessels/mm^2^ after analyzing three high-power fields (400 ×).

### Analysis of the hematological responses to HF in vivo

At day 21 mice were sacrificed after being subjected to a cardiac puncture to obtain peripheral blood [36] samples. Age-matched NOD/SCID healthy animals were used as controls (WT; *n* = 3). Automated counts for white blood cells (WBC) were performed using a T-890 Coulter cell counter (Coulter Corporation, Hialeah, FL, USA), and differential counts were obtained from Leishman-Wright-Giemsa-stained smears. For morphological analysis, 100 cells of PB were counted, and then myeloid cells were classified as immature, intermediate, or mature, according to the Bethesda criteria [37]. Bone marrow (BM) cells were obtained by flushing of the bone cavities and the cells were used for cytospin slide preparation and staining with Leishman-Wright-Giemsa. Imprint slides of the spleen were also performed to quantify the hematological response to HF.

### Immnunophenotyping of bone marrow cells

BM cells were characterized by immnunophenotyping using the following combinations of mAbs: CD117-PE/CD16/32-FITC/CD45-Per-CP; CD11b-PE/Gr-1-FITC/CD45-PerCP. Fluorochrome-conjugated isotypic antibodies were used as negative controls. All antibodies were purchased from BD Biosciences Pharmingen (San Diego, CA, USA). A minimum of 10,000 events/tube were acquired with a FACScalibur flow cytometer. Viable CD45-positive cells were gated and the percentage of each cell subset was determined using the CellQuest software (Becton Dickinson).

### ChIP assay

NB4 cells were stimulated with Halofuginone (200 ng/mL), TGF-β (50 ng/ml) or Halofuginone (200ng/mL) + TGF-β (50ng/mL), for 6 hours and the chormatin immunoprecipitation was performed with Magna ChIP™ G (Millipore, Billerica, MA, USA) according to the instructions of manufacturer. Briefely, the crosslink was made with 1 % formaldehyde followed by addition of 125mM glicine to quench unreacted formaldehyde. Samples were then resuspended in cell lysis buffer containing Proteases Inhibitors before addition of Nuclear Lysis Buffer. The final lysates were sonicated to shear the genomic DNA into around 200-1000pb fragments. ChIP assay was performed on the supernatants by addition of 5 μg of each antibody: Anti-P-Smad2 (phospho S467 - ab192191); Anti-Smad2 + Smad3 (ab63672); Anti-HIF-1α (ab2185) and IgG rabbit polyclonal (ab27478). Primers for Vegf promoter region were custom designed (Additional file 1: Table S1). Graphics show Fold enrichment over IgG antibodie control for each sample and primer. Primers were purchased from Sigma- Aldrich (St. Louis, MO, USA).

### Statistical analysis

All experiments were evaluated by analysis of the variance (ANOVA) between groups followed by the Bonferroni’s correction post-test or Mann-Whitney unpaired t-test. In all comparisons, a significance level of *P* < 0.05 was considered to be significant. Statistical analyses were performed using SPSS 13.0 software.

## Results

### Angiogenesis is aberrantly increased in the APL transgenic mouse model

**Fig. 1:**
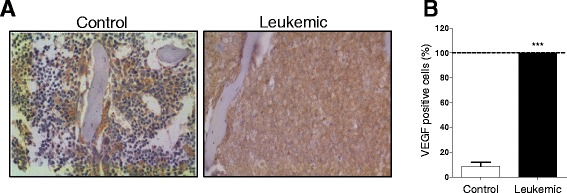
Expression of the pro-angiogenic mediator VEGF is increased in APL transgenic mouse model. **a** Bone marrow (BM) samples of non-leukemic control hCG-PML-RARα transgenic mice (TM) and hCG-PML-RARα transgenic mice at leukemia diagnosis. **b** Percentage of VEGF positive cells detected by VEGF-staining by immunohistochemistry and analysis of three microscopic fields (400×) (in brown). ***P < 0.0001 (t-test)

Based on the demonstration of high levels of VEGF in BM biopsies from APL patients [15], we first investigated VEGF expression in an APL transgenic mouse (TM) model expressing the PML/RARα fusion gene under the control of human cathepsin gene promoter (hCG) [33, 38]. Fig. 1a and b show the immunohistochemistry analysis of VEGF expression in leukemic and control BM TM samples. There was a striking increase in the number VEGF positive cells in leukemic BM and, by correlating with H&E staining (not shown), it was possible to identify that most VEGF positive cells were leukemic blasts. In addition, the number of microvessels/mm^2^ in BM sections of leukemic TM was higher than that of controls (mean of 65.2 ± 38.44 vs. 18.25 ± 7.66 microvessels/mm^2^, respectively). These results demonstrate that the PML-RARα TM model reproduces the increased MVD and VEGF reported in human APL.

### Halofuginone modulates expression of genes associated with angiogenesis

**Fig. 2:**
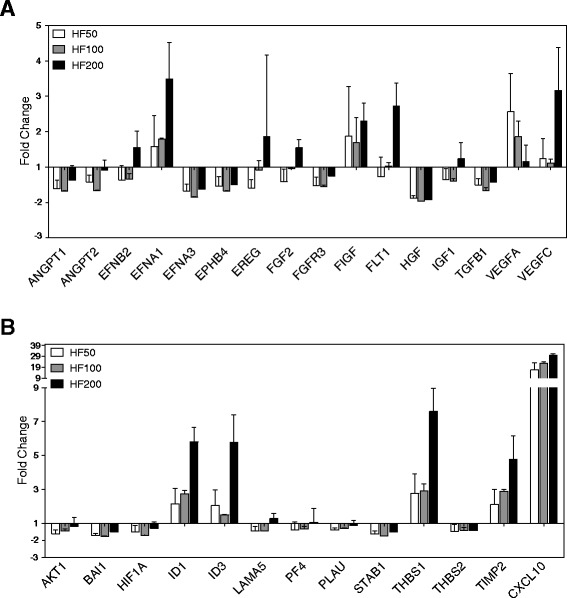
Gene expression of angiogenic factors is down-modulated by halofuginone. **a**, **b** NB4 cells were treated with 50, 100 or 200 ng/mL of HF for 6 hours and mRNA expression of angiogenesis determinants was assessed by PCR array. Graphic shows genes with greater differential expression compared to nontreated cells (Fold change = 1). Data are representative of two independent experiments (error bars, s.e.m.)

Jordan and Zeplin, showed that HF significantly reduces the gene expression of pro-angiogenic factors in physiologic conditions in muscle cells [25]. In order to evaluate the effect of HF in leukemic cells, we treated the APL cell line NB4 and performed a screening of gene expression using the PCR array method analyzing 84 predefined genes involved in angiogenesis (Additional file 2: Table S2). Among the 40 genes whose expression was modulated by incubation with HF at the dose of 50ng/ml for 6 hours, 16 encoded for growth factors and receptors, 6 for cytokines, 5 for proteases inhibitors and matrix proteins, 4 for adhesion molecules and 3 for transcription factors. As highlighted in Fig. 2a and b the expression of most proangiogenic factors was decreased and the antiangiogenic factors TIMP2 and CXCL10 was increased at all HF concentrations. The largest differences were noted for HIF-1α (mean of 0.48 ± 0.33 fold change), HGF (0.12 ± 0.056), angiopoetin-1 (ANGPT-1: 0.38 ± 0.19), angiopoetin-2 (ANGPT-2: 0.57 ± 0.16), TIMP2 (2.1 ± 0.74) and CXCL10 (16.5 ± 5.4). HF dose dependent effect was detected for VEGFA, TIMP2 and CXCL10.

**Fig. 3:**
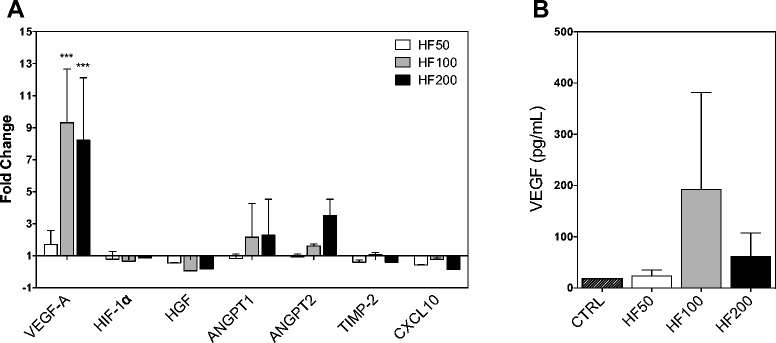
HF effects on the angiogenic factors in endothelial cell HUVEC. **a** Cells were treated with 50, 100 or 200 ng/mL of HF for 24 hours and mRNA expression of angiogenic factors was assessed by real time PCR. Graphic shows differential expression compared to non-treated cells (Fold change = 1). **b** Cells were treated for 72 hours to quantify VEGF production in the supernatant. CTRL: non-treated cells. ***P < 0.0001; (one-way ANOVA). Data are representative of two independent experiments (error bars, s.e.m.)

We next evaluate if the HF effects over gene expression would extend to endothelial cells. Interestingly, HUVEC cells treated with the aforementioned HF concentrations, showed increased VEGF gene expression (9.3 ± 3.34; at 100 ng/mL) and protein secretion (Fig. 3a and 3b). Moreover, ANGPT-1, HIF-1α, HGF, TIMP2 and CXCL10 did not show significant change in gene expression while ANGPT-2 showed increased expression (3.5 ± 1.02; at 200 ng/mL).

### Halofuginone inhibits pro-angiogenic mediators in the APL cell line NB4

**Fig. 4:**
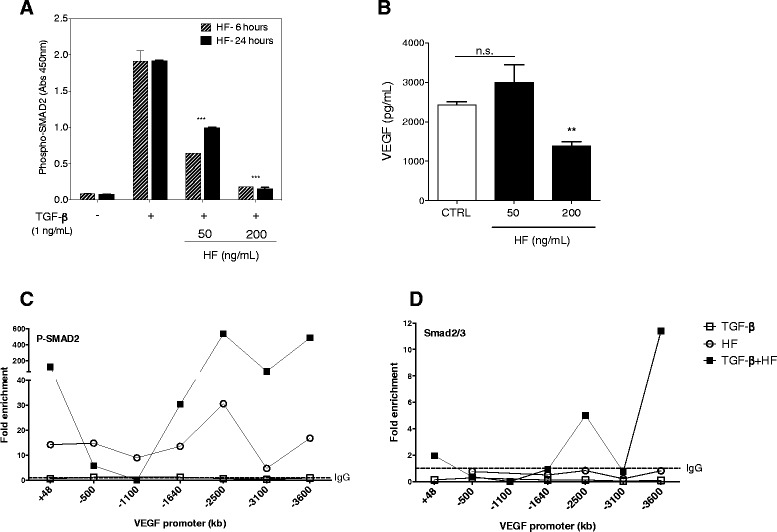
Halofuginone inhibits pro-angiogenic mediators in NB4 cells. **a** Phospho-SMAD2 identification assessed by ELISA in NB4 cells treated with 50 and 200 ng/mL of HF for 6 and 24 hours hours and TGF-β (1ng/mL) was added to the cultures 1 hour before. **b** Concentration of VEGF determined by CBA in the supernatant of NB4 cells treated with 50 and 200 ng/mL doses of HF during 72 hours. CTRL: non-treated cells. **c**, **d** ChIP assay using antibodies to P-Smad2 and Smad2/3. NB4 cells were treated with 200 ng/mL of HF for 6 hours, or treated with 200 ng/mL of HF for 6 hours and stimulated with TGF-β (1ng/mL) in the last hour, or only stimulated with TGF-β. Graphics showing a PCR amplification of ChIP products using a series of primer pairs covering the upstream and downstream the transcription start site (TSS) of Vegf. Data is represented as fold enrichment to IgG control antibody. **P < 0.001, ***P < 0.0001; (one-way ANOVA). Data are representative of two **a** and three **b**) independent experiments (error bars, s.e.m.)

Previously, we have demonstrated that HF at concentrations higher than 75 ng/ml induced TGF-β and TGFβ-R2 protein expression in NB4 cells [1]. TGF-β-cell responses are coordinated through SMADs phosphorylation, which translocate to nucleus and work as transcription factors [39]. Thus, we further analyzed the phosphorylation of SMAD2 in NB4 cells. HF treatment for 6 and 24 hours impaired phosphorylation of SMAD2 (Fig. 4a), which was a dose- and time-dependent effect that lasted at least for 24 hours. Based on the fact that the promoter region of the VEGF gene contains DNA-binding sequences for SMADs [27, 28] and the previous report that the NB4 cells secretes high levels of VEGF [15], we evaluated the effect of HF treatment in NB4 VEGF production. Quantification of cell-culture supernatants of NB4 cells treated with HF for 72 hours showed that VEGF levels were strongly reduced using HF concentration of 200 ng/mL. (Fig. 4b). In order to verify if SMAD2 has a role as transcription factor for VEGF expression in NB4 cells, we performed a ChIP assay using antibodies for the phosphorylated form of SMAD2 (P-SMAD2) and for the heterodimer SMAD2/3. Analysis of approximately 3,700 pb neighboring the transcription start site (TSS) of VEGF gene showed that, NB4 stimulation with TGF-β increases P-SMAD2 binding in several sites upstream and downstream VEGF. HF treatment prior TGF-β stimulation enhanced P-SMAD2 (Fig. 4c). On the other hand, ChIP assay using anti-SMAD2/3 did not showed binding of unphosphorylated SMADs upon stimulation with TGF-β, however the previous treatment with HF showed increase increase in two sites (2,500 and 3,600 upstream TSS) (Fig. 4d). Similarly, HIF-1α binding was not induced only by TGF-β stimulation, and was enhanced with HF treatment (Additional file 3: Figure S1A). Anti-RNA Polimerase II was used as a control of the ChIP assay (Additional file 3: Figure S1B), and showed increased binding in all the sites analyzed upon TGF-β stimulation with or without HF treatment.

### Halofuginone displays an anti-leukemic effect in the in vivo model of APL

**Fig. 5:**
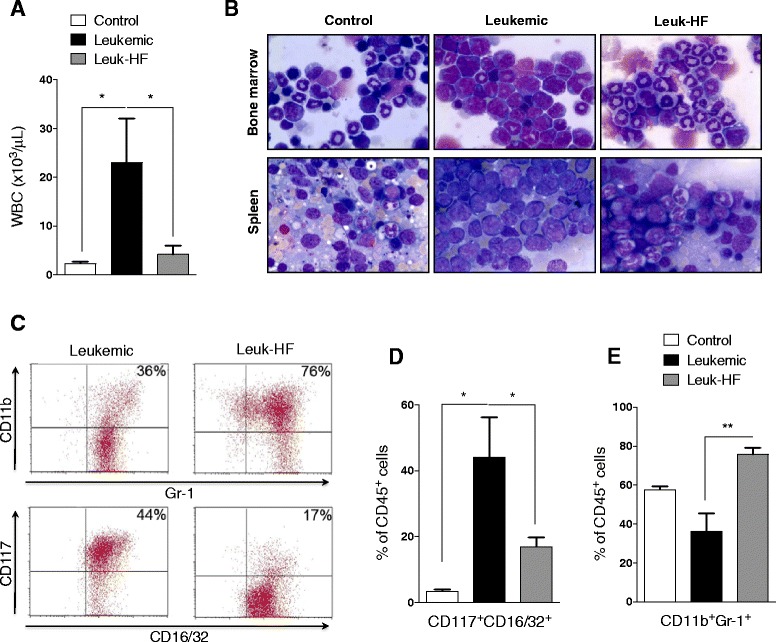
Halofuginone displays an anti-leukemic effect in the *in vivo* model of APL. **a** WBC account in peripheral blood obtained non-leukemic (control), leukemic and leukemic mice treated for 21 days with HF (Leuk-HF). **b** BM-cell spin and spleen imprints from control and leukemic mice. Leishman-Wright-Giemsa staining. **c** Immnunophenotyping of BM cells from leukemic and leuk-HF. **d** CD117^+^CD16/32^+^ characterize murine promyelocytes and **e** CD11b^+^Gr-1^+^ are double expressed in more differentiated cells

**Fig. 6:**
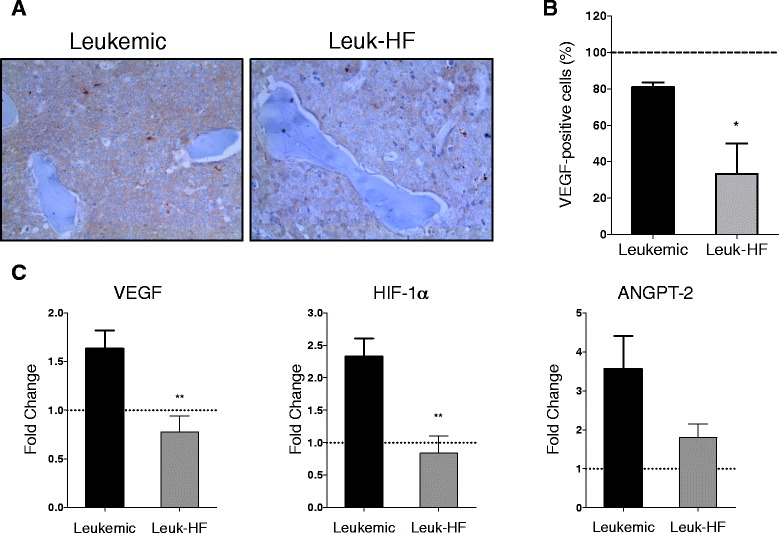
Antiangiogenic effects of HF in the *in vivo* model of APL. **a** Immunohistochemistry staining for VEGF in BM samples of leukemic and leukemic mice treated for 21 days with HF (Leuk-HF). **b** Percentage of VEGF positive cells detected by VEGF-staining by immunohistochemistry and analysis of three microscopic fields (400×). **c** mRNA expression in BM samples of leukemic and Leuk-HF mice. Doted line indicates non-leukemic mice (Fold change = 1). *P < 0.05, **P < 0.001 (one-way ANOVA). Data summarize one representative experiment (*n* = 5) (error bars, sd)

To investigate whether the anti-angiogenic properties of HF resulted in anti-leukemic effects in APL, we used a transplant model of PML-RARα leukemic cells in NOD/SCID mice. All recipients developed extensive BM and spleen infiltration, leukocytosis and succumbed of the disease within 30-40 days [1]. This strategy was necessary due to the long latency and low penetrance of the transgenic model, which require the observation of a large cohort of TM. Therefore, recipient mice were treated with HF i.p. for 21 days starting on Day 2 post transplant and at the end of treatment mice were sacrificied. Treatment with HF significantly reduced white blood cell (WBC) counts in leukemic mice (Fig. 5a). [33]. Morphological analysis of Leishman-Wright-Giemsa-stained slides of BM and spleen imprints confirmed the infiltration of immature cells in leukemic animals and showed a significant reduction of this cell pattern in the HF-treated mice (Fig. 5b). The immunophenotypic analysis of BM showed that HF-treated mice had a higher percentage of more differentiated CD11b^+^/Gr-1^+^ myeloid cells and lower of CD117^+^CD16/32^+^ (previously defined as murine leukemic cells [40]) compared to leukemic non-treated mice (Fig. 5c, d and e). Finally, in addition to the anti-proliferative action, the reduction of leukemia burden observed with HF treatment correlated with antiangiogenic effects. Quantitative and qualitative expression of VEGF by immunohistochemistry showed a reduced number of VEGF^+^ cells and lower VEGF expression in BM sections from HF-treated mice than in non-treated control (Fig. 6a). Likewise, in our findings with the leukemic PML-RARα TM, most of the VEGF positive cells corresponded to immature cells (Fig. 6b). Nevertheless, the reduction of the MVD in HF-treated mice did not reach statistical significance (158 ± 32.5 vs. 118 ± 35 microvessels/mm^2^ in controls (*n* = 5) and treated leukemic mice (*n* = 5), respectively). In the other hand, PCR analysis of BM samples also showed reduction in VEGF, HIF-1α and ANGPT-2 gene expression in HF-treated mice (Fig. 6c), whereas ANGPT-1, HGF, TIMP2 and CXCL10 had no change in leukemic mice (data not shown).

## Discussion

Here we report that HF reduced tumor burden in an APL mouse model as showed by the cytomorphological and immunophenotyping analysis of the BM after 21 days of treatment. This is the first study to show that leukemic hCG-PML/RARA TM had increased VEGF expression in BM, reinforcing that VEGF may be a mediator supporting leukemogenesis [41]. Using a transplant model of PML-RARα leukemic cells, the treatment with HF reduced the number of VEGF^+^ cells and VEGF gene expression in BM of leukemic mice. However, HF treatment did not result in a significant reduction of MVD. This may be a consequence of the transplant model used, which led to a massive infiltration of the BM with a shorter survival compared to solid tumor models. In this regard it is worthy pointing out that in the study by Elkin et al. the animals with bladder cancer were treated with oral HF for 26 and 29 weeks [42].

In addition, the treatment of the endothelial cell HUVEC with HF did not change the gene expression of ANGPT-1, HIF-1α, TIMP2 and CXCL10, whereas increased VEGF gene expression and protein secretion. These data suggests that HF effects may present variation according to the cell type, which is in accord with the previous demonstrated increase in VEGF production by fibrotic liver cells from rats treated with HF [43]. Furthermore, it is well established that tumor cells-derived VEGF stimulates endothelial cells to produce VEGF [44]. Considering that the basal production of VEGF showed by NB4 cells is over 100-fold higher, the induction of VEGF by HF treatment in endothelial cells may not have impact in the angiogenesis process.

In non-APL AML Schuch et al. [45] demonstrated that VEGF administration resulted in enhanced tumor growth and vascularization whereas treatment with a VEGF antagonist soluble NRP-1 inhibited tumor angiogenesis and growth in a chloroma mouse model. Moreover, these authors showed that the injection of adenovirus encoding NRP-1 reduced the leukemic burden and prolonged the survival of leukemic mice. Recently, Oka et al. described a high level of VEGF-A in the serum of an APL patient at diagnosis and the ex-vivo treatment with an anti-VEGF-A reduced leukemic cell proliferation [46]. Unfortunately, in the phase II HOVON/SAKK AML study, the administration of the anti-VEGF agent bevacizumab along with standard chemotherapy did not result in better outcome in older patients [47]. Thus, despite experimental results indicating that VEGF have a potential therapeutic benefit, the HOVON/SAKK study suggests that inhibition of VEGF may not be sufficient to control angiogenesis or tumor growth.

Considering HF antiangiogenic properties, we used a PCR array to screening angiogenic mediators in the APL cell line NB4. Our data showed a down-regulation mediated by HF in several pro-angiogenic factors, including HFG, HIF-1α, ANGPT-1 and -2, whereas the anti-angiogenic factors TIMP2 and CXCL10 were over expressed. Indeed, HGF is a well-recognized proangiogenic mediator which inhibition reduces tumor growth and metastasis [48], as well HIF-1α, which is associated with poor prognosis in cancer due stimulation of VEGFR^+^ cells migration to the tumor [49]. Contrary, CXCL10 and TIMP2 had they antiangiogenic potential demonstrated. Mice with fibrosarcoma treated with CXCL10 combined with hypothermia presented reduced tumor burden and MVD [50], whereas the MMP2-inhibitor TIMP2, have been recently described as important prognostic factors in acute lymphoblastic leukemia [51]. In the leukemia mouse model, we did observe increased gene expression of VEGF, HIF-1α and ANGPT-2, which were significantly reduced after HF treatment. These PCR data support that HF antiangiogenic effect implicates multiple targets, as demonstrated by Jordan ans Zeplin [25], MMP-2, bFGF and TGF-β were down-modulated by HF in physiologic conditions.

On the other hand, addition of HF to NB4 cells cultures did not result in morphological differentiation [1] likewise, no differentiation was observed in BM and blood smears of recipient mice at seven and 14 days of HF treatment (data not shown). Therefore, we believe that increase in differentiated cells in BM of HF treated mice reflects the restoration of normal hematopoiesis secondary to the significant reduction of the leukemic infiltration. Together, our results corroborate with previous studies reporting the anti-proliferative and proapoptotic actions of HF in solid tumors [32, 52] and in leiomyoma cell line [53] and with recent observations from our group demonstrating that HF induced the blockade of cell cycle progression at G1/S transition and apoptosis in NB4 cells [1]. Here we showed that NB4 cells treated with HF had SMAD2 phosphorylation abrogated indicating that the TGF-β-signaling was inhibited, which is in agreement with previous studies [54–56]. It is proposed that VEGF gene expression is targeted by TGF-β- signaling [57–59] and that low concentration of TGF-β cooperate with VEGF to stimulate epithelial cell proliferation and migration [60]. Thus, one possible explanation for the VEGF reduction induced by HF is the inhibition of the TGF-β-signaling. One result suggest that HF acts by decreasing SMAD-2 phosphorylation triggered by TGF-β. However, ChIP analysis showed that, despite reduction of SMAD phosphorylation, HF treatment increases P-SMAD2 binding to VEGF promoter region. Importantly, it has been demonstrated that SMAD2 is not required for VEGF gene expression, and that SMAD3 is the potential transcription factor linking TGF-β-signaling and VEGF [57].

It has been proposed that the PML-RARα fusion protein, similarly to cPML interacts and co-localizes with SMAD2/3 and SARA thus interfering with the phosphorylation and nuclear translocation of this complex Lin et al. [26]. The authors hypothesized that PML/RARα in the cytosol inhibits TGF-β-signaling by sequestering cPML away from the TFβR1/SARA/SMAD2/3 complex thus preventing the trafficking to the early endosome. This inhibition could be further enhanced by interfering with nuclear isoform PML4, which has been demonstrated to transactivate TGF-β target genes. Our results showed that NB4 cells responded to the addition of TGF-β in culture medium with an increase of about 20-fold in SMAD2 phosphorylation. HF was capable of inhibiting TGF-β-induced SMAD2 phosphorylation. Taken together with our previous report that HF blocked NB4 proliferation and reduced the leukemia burden *in vivo* we postulated that despite the interference of PML/RARα with TFβR1/SARA/SMAD2/3 complex, the TGF-β pathway remain active in APL and contributes for leukemia progression.

## Conclusion

Here we showed HF can affect multiply mechanism and that HF treatment reduced proangiogenic factors expression and TGF-β-signaling *in vitro,* in addition to displaying antiangiogenic and antileukemic effects in an animal model of APL. Considering the importance of angiogenesis and deregulation of TGF-β-signaling in tumor progression, we propose HF as a potential new anti-cancer drug

## Additional files

Additional file 1: Table S1. Vegf primers used for ChIP assay. Additional file 2: Table S2. List of angiogenesis-related genes assessed by PCR array. Additional file 3: Figure S1. ChIP assay using antibodies to RNA Polimerase II, Smad2/3, P-Smad2 and HIF-1α. NB4 cells were treated with 200 ng/mL of HF for 6 hours, or treated with 200ng/mL of HF for 6 hours and stimulated with TGF-β (1ng/mL) in the last hour, or only stimulated with TGF-β. Graphics showing a PCR amplification of ChIP products for (A) HIF-1α and (B) RNA Polimerase II antibodies using a series of primer pairs covering the Vegf promoter. Data is represented as fold enrichment to IgG control antibody.
