# Supplementary material for: Halofuginone inhibits phosphorylation of SMAD-2 reducing angiogenesis and leukemia burden in an acute promyelocytic leukemia mouse model
Source: J Exp Clin Cancer Res. 2015 Jun 23;34(1):65. doi: 10.1186/s13046-015-0181-2 (PMC4486128; doi:10.1186/s13046-015-0181-2)
Supplement: Additional file 1: Table S1. — Vegf primers used for ChIP assay. [file 13046_2015_181_MOESM1_ESM.docx]

Additional file 1: Table S1. *Vegf* primers used for ChIP assay

| Upstream the Start Transcription site of VEGF | |
| --- | --- |
| -500_F | 5’-CTTTAGCCAGAGCCGGGGTGTGC-3’ |
| -500_R | 5’-AAGACGCTGCTCGCTCCATTCA-3’ |
| -1100_F | 5’-GGAGGGGCTGGGGGAAGGATAGG-3’ |
| -1100_R | 5’-TCAGCCCAAGCCCAGACTCATAGC-3’ |
| -1640_F | 5’-AGGGCCTTAGGACACCATACC3’ |
| -1640_R | 5’-TAGTCAGCCCCATCCTCAGCACAT-3’ |
| -2500_F | 5’-CTGGGGAGGGAGGTGAGGATAAG-3’ |
| -2500_R | 5’-AAGGGGAAAAAGCACAAAACAACA-3’ |
| -3100_F | 5’-TGGGGGTCAGGGGATGGAGT-3’ |
| -3100_R | 5’-AGCCGGTGGATGAAGGGACAAAAT-3’ |
| -3600_F | 5’-GCTGATGGGCCTAGGGGTGGTG-3’ |
| -3600_R | 5’-CAGTCAGGAAAGGGGGAAGGAATG-3’ |
| Downstream the Start Transcription site of VEGF | |
| +48_F | 5’-CTCCCGCCCCCTTTCCCTCTTC-3’ |
| +48_R | 5’-GGTCACCCCATATCCTACACATTA-3’ |
