# Supplementary material for: Halofuginone inhibits phosphorylation of SMAD-2 reducing angiogenesis and leukemia burden in an acute promyelocytic leukemia mouse model
Source: J Exp Clin Cancer Res. 2015 Jun 23;34(1):65. doi: 10.1186/s13046-015-0181-2 (PMC4486128; doi:10.1186/s13046-015-0181-2)
Supplement: Additional file 2: Table S2. — List of angiogenesis-related genes assessed by PCR array. [file 13046_2015_181_MOESM2_ESM.doc]

| **Symbol** | **GeneBank** | **Fold Change*** | **Description** |
| --- | --- | --- | --- |
| **AKT1** | NM_005163 | 0.384 | V-akt murine thymoma viral oncogene homolog 1 |
| **ANG** | NM_001145 | 0.386 | Angiogenin, ribonuclease, RNase A family, 5 |
| **ANGPT1** | NM_001146 | 0.576 | Angiopoietin 1 |
| **ANGPT2** | NM_001147 | ----- | Angiopoietin 2 |
| **ANGPTL4** | NM_001039667 | 0.758 | Angiopoietin-like 4 |
| **ANPEP** | NM_001150 | 0.730 | Alanyl (membrane) aminopeptidase |
| **BAI1** | NM_001702 | 0.277 | Brain-specific angiogenesis inhibitor 1 |
| **CCL11** | NM_002986 | 1.493 | Chemokine (C-C motif) ligand 11 |
| **CCL2** | NM_002982 | 0.674 | Chemokine (C-C motif) ligand 2 |
| **CDH5** | NM_001795 | ----- | Cadherin 5, type 2 (vascular endothelium) |
| **COL18A1** | NM_030582 | 1.008 | Collagen, type XVIII, alpha 1 |
| **COL4A3** | NM_000091 | 0.992 | Collagen, type IV, alpha 3 (Goodpasture antigen) |
| **CTGF** | NM_001901 | 2.326 | Connective tissue growth factor |
| **CXCL1** | NM_001511 | 16.543 | Chemokine (C-X-C motif) ligand 1 |
| **CXCL10** | NM_001565 | 4.018 | Chemokine (C-X-C motif) ligand 10 |
| **CXCL5** | NM_002994 | 0.507 | Chemokine (C-X-C motif) ligand 5 |
| **CXCL6** | NM_002993 | 0.985 | Chemokine (C-X-C motif) ligand 6 |
| **CXCL9** | NM_002416 | 0.418 | Chemokine (C-X-C motif) ligand 9 |
| **EDN1** | NM_001955 | 1.026 | Endothelin 1 |
| **EFNA1** | NM_182685 | 1.062 | Ephrin-A1 |
| **EFNB2** | NM_004093 | 1.582 | Ephrin-B2 |
| **EGF** | NM_001963 | 0.326 | Epidermal growth factor |
| **ENG** | NM_000118 | 0.637 | Endoglin |
| **EPHB4** | NM_004444 | 1.766 | EPH receptor B4 |
| **ERBB2** | NM_004448 | 0.921 | V-erb-b2 erythroblastic leukemia viral oncogene |
| **F3** | NM_001993 | 0.462 | Coagulation factor III (thromboplastin, tissue factor) |
| **FGF1** | NM_000800 | 0.410 | Fibroblast growth factor 1 (acidic) |
| **FGF2** | NM_002006 | 1.171 | Fibroblast growth factor 2 (basic) |
| **FGFR3** | NM_000142 | 0.594 | Fibroblast growth factor receptor 3 |
| **FIGF** | NM_004469 | 0.477 | C-fos induced growth factor |
| **FLT1** | NM_002019 | 1.869 | Fms-related tyrosine kinase 1 |
| **FN1** | NM_002026 | 0.723 | Fibronectin 1 |
| **HGF** | NM_000601 | 0.978 | Hepatocyte growth factor |
| **HIF1A** | NM_001530 | 0.127 | Hypoxia inducible factor 1, alpha subunit |
| **HPSE** | NM_006665 | 0.485 | Heparanase |
| **ID1** | NM_002165 | 1.061 | Inhibitor of DNA binding 1 |
| **IFNA1** | NM_024013 | 2.154 | Interferon, alpha 1 |
| **IFNG** | NM_000619 | 2.050 | Interferon, gamma |
| **IGF1** | NM_000618 | 0.639 | Insulin-like growth factor 1 (somatomedin C) |
| **IL1B** | NM_000576 | 1.456 | Interleukin 1, beta |
| **IL6** | NM_000600 | ----- | Interleukin 6 (interferon, beta 2) |
| **IL8** | NM_000584 | 0.642 | Interleukin 8 |
| **ITGAV** | NM_002210 | 1.554 | Integrin, alpha V |
| **ITGB3** | NM_000212 | 0.846 | Integrin, beta 3 |
| **JAG1** | NM_000214 | 0.839 | Jagged 1 |
| **KDR** | NM_002253 | 0.942 | Kinase insert domain receptor |
| **LECT1** | NM_007015 | 1.263 | Leukocyte cell derived chemotaxin 1 |
| **LEP** | NM_000230 | 1.810 | Leptin |
| **MDK** | NM_002391 | 0.808 | Midkine (neurite growth-promoting factor 2) |
| **MMP14** | NM_004995 | 0.571 | Matrix metallopeptidase 14 (membrane-inserted) |
| **MMP2** | NM_004530 | 0.569 | Matrix metallopeptidase 2 |
| **MMP9** | NM_004994 | 1.413 | Matrix metallopeptidase 9 |
| **NOS3** | NM_000603 | 0.973 | Nitric oxide synthase 3 (endothelial cell) |
| **NOTCH4** | NM_004557 | 0.723 | Notch 4 |
| **NRP1** | NM_003873 | 0.694 | Neuropilin 1 |
| **NRP2** | NM_003872 | 1.329 | Neuropilin 2 |
| **PDGFA** | NM_002607 | 0.839 | Platelet-derived growth factor alpha polypeptide |
| **PECAM1** | NM_000442 | ----- | Platelet/endothelial cell adhesion molecule |
| **PF4** | NM_002619 | 1.086 | Platelet factor 4 |
| **PGF** | NM_002632 | 0.788 | Placental growth factor |
| **PLAU** | NM_002658 | 0.623 | Plasminogen activator, urokinase |
| **PLG** | NM_000301 | 0.861 | Plasminogen |
| **PROK2** | NM_021935 | 0.601 | Prokineticin 2 |
| **PTGS1** | NM_000962 | 0.676 | Prostaglandin-endoperoxide synthase 1 |
| **S1PR1** | NM_001400 | 1.218 | Sphingosine-1-phosphate receptor 1 |
| **SERPINE1** | NM_000602 | 0.296 | Serpin peptidase inhibitor, clade E, member 1 |
| **SERPINF1** | NM_002615 | 0.403 | Serpin peptidase inhibitor, clade F |
| **SPHK1** | NM_021972 | 1.676 | Sphingosine kinase 1 |
| **TEK** | NM_000459 | 0.838 | TEK tyrosine kinase, endothelial |
| **TGFA** | NM_003236 | 0.377 | Transforming growth factor, alpha |
| **TGFB1** | NM_000660 | 0.885 | Transforming growth factor, beta 1 |
| **TGFB2** | NM_003238 | 3.979 | Transforming growth factor, beta 2 |
| **TGFBR1** | NM_004612 | 0.492 | Transforming growth factor, beta receptor 1 |
| **THBS1** | NM_003246 | 0.691 | Thrombospondin 1 |
| **THBS2** | NM_003247 | 0.794 | Thrombospondin 2 |
| **TIE1** | NM_005424 | 2.780 | Tyrosine kinase with immunoglobulin-like domains 1 |
| **TIMP1** | NM_003254 | 0.525 | TIMP metallopeptidase inhibitor 1 |
| **TIMP2** | NM_003255 | 0.788 | TIMP metallopeptidase inhibitor 2 |
| **TIMP3** | NM_000362 | 2.103 | TIMP metallopeptidase inhibitor 3 |
| **TNF** | NM_000594 | 1.163 | Tumor necrosis factor |
| **TYMP** | NM_001953 | 0.663 | Thymidine phosphorylase |
| **VEGFA** | NM_003376 | 0.646 | Vascular endothelial growth factor A |
| **VEGFB** | NM_003377 | 2.563 | Vascular endothelial growth factor B |
| **VEGFC** | NM_005429 | 1.233 | Vascular endothelial growth factor C |
| **ACTB** | NM_001101 | 0.384 | Actin, beta |
| **B2M** | NM_004048 | 0.386 | Beta-2-microglobulin |
| **GAPDH** | NM_002046 | 0.576 | Glyceraldehyde-3-phosphate dehydrogenase |
| **HPRT1** | NM_000194 | ----- | Hypoxanthine phosphoribosyltransferase 1 |
| **RPLP0** | NM_001002 | 0.758 | Ribosomal protein, large, P0 |
| **HGDC** | SA_00105 | 0.730 | Human Genomic DNA Contamination |
| **RTC** | SA_00104 | 0.277 | Reverse Transcription Control |
| **RTC** | SA_00104 | 1.493 | Reverse Transcription Control |
| **RTC** | SA_00104 | 0.674 | Reverse Transcription Control |
| **PPC** | SA_00103 | ----- | Positive PCR Control |
| **PPC** | SA_00103 | 1.008 | Positive PCR Control |
| **PPC** | SA_00103 | 0.992 | Positive PCR Control |
