# Supplementary material for: Halofuginone inhibits phosphorylation of SMAD-2 reducing angiogenesis and leukemia burden in an acute promyelocytic leukemia mouse model
Source: J Exp Clin Cancer Res. 2015 Jun 23;34(1):65. doi: 10.1186/s13046-015-0181-2 (PMC4486128; doi:10.1186/s13046-015-0181-2)
Supplement: Additional file 3: Figure S1. — ChIP assay using antibodies to RNA Polimerase II, Smad2/3, P-Smad2 and HIF-1α. NB4 cells were treated with 200 ng/mL of HF for 6 hours, or treated with 200ng/mL of HF for 6 hours and stimulated with TGF-β (1ng/mL) in the last hour, or only stimulated with TGF-β. Graphics showing a PCR amplification of ChIP products for (A) HIF-1α and (B) RNA Polimerase II antibodies using a series of primer pairs covering the Vegf promoter. Data is represented as fold enrichment to IgG control antibody. [file 13046_2015_181_MOESM3_ESM.pdf]

Supplementary Figure - S1

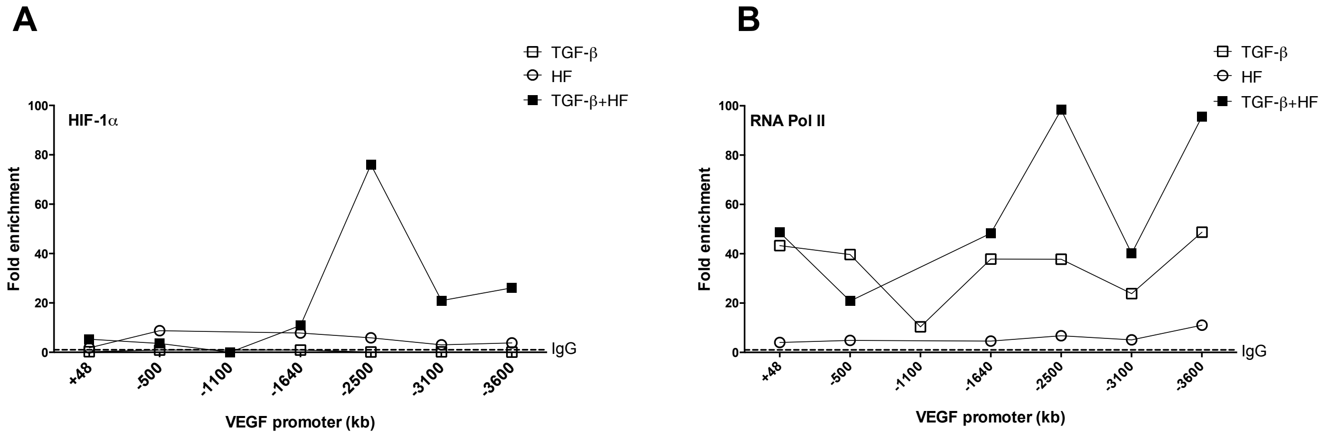

Figure S1 - ChIP assay using antibodies to RNA Polimerase II, Smad2/3, P-Smad2 and HIF-1α. NB4 cells were treated with 200 ng/mL of HF for 6 hours, or treated with 200 ng/mL of HF for 6 hours and stimulated with TGF-β (1ng/mL) in the last hour, or only stimulated with TGF-β. Graphics showing a PCR amplification of ChIP products for (A) HIF-1α and (B) RNA Polimerase II antibodies using a series of primer pairs covering the *Vegf* promoter. Data is represented as fold enrichment to IgG control antibody.
